# Plasma levels of oxidative stress-responsive apoptosis inducing protein (ORAIP) in rats subjected to physicochemical oxidative stresses

**DOI:** 10.1042/BSR20160044

**Published:** 2016-04-15

**Authors:** Takako Yao, Tsutomu Fujimura, Kimie Murayama, Yoshinori Seko

**Affiliations:** * Division of Cardiovascular Medicine, The Institute for Adult Diseases, Asahi Life Foundation, 2-2-6 Nihonbashi-Bakurocho, Chuo-ku, Tokyo 103-0002, Japan; † Laboratory of Bioanalytical Chemistry, Tohoku Pharmaceutical University, 4-4-1 Komatsushima, Aoba-ku, Sendai, Miyagi 981-8558, Japan; ‡ Division of Proteomics and Biomolecular Science, BioMedical Research Center, Graduate School of Medicine, Juntendo University, Bunkyo-ku, Tokyo 113-8421, Japan

**Keywords:** acidification, eukaryotic translation initiation factor 5A, heat shock, humoral factor, oxygenation

## Abstract

Oxidative stress is known to play a pivotal role in the pathogenesis of various disorders including atherosclerosis, aging and especially ischaemia/reperfusion injury. It causes cell damage that leads to apoptosis. However, the precise mechanism has been uncertain. Recently, we identified an apoptosis-inducing humoral factor in a hypoxia/reoxygenated medium of cardiac myocytes. We named this novel post-translationally modified secreted form of eukaryotic translation initiation factor 5A (eIF5A) as oxidative stress-responsive apoptosis inducing protein (ORAIP). We developed a sandwich ELISA and confirmed that myocardial ischaemia/reperfusion markedly increased plasma levels of ORAIP. To investigate whether the role of ORAIP is common to various types of oxidative stress, we measured plasma ORAIP levels in rats subjected to three physicochemical models of oxidative stress including N_2_/O_2_ inhalation, cold/warm-stress (heat shock) and blood acidification. In all three models, plasma ORAIP levels significantly increased and reached a peak level at 10–30 min after stimulation, then decreased within 60 min. The (mean±S.E.M.) plasma ORAIP levels before and after (peak) stimulation were (16.4±9.6) and (55.2±34.2) ng/ml in N_2_/O_2_ inhalation, (14.1±12.4) and (34.3±14.6) ng/ml in cold/warm-stress, and (18.9±14.3) and (134.0±67.2) ng/ml in blood acidification study. These data strongly suggest that secretion of ORAIP in response to oxidative stress is universal mechanism and plays an essential role. ORAIP will be an important novel biomarker as well as a specific therapeutic target of these oxidative stress-induced cell injuries.

## Abstract (precis)

Plasma levels of a novel oxidative stress-responsive apoptosis inducing protein (ORAIP) were significantly increased in rats subjected to three physicochemical models of oxidative stress indicating that the response is specific to oxidative stress playing a critical role in the pathogenesis involved.

## INTRODUCTION

Oxidative stress plays a pivotal role in ischaemia/reperfusion injury, atherosclerosis, aging and so on. It causes cell damage that leads to apoptosis; however, the precise mechanism has been unclear. Using an *in vitro* model of myocardial ischaemia/reperfusion, we analysed the molecular mechanism involved in hypoxia/reoxygenation-induced apoptosis of cultured cardiac myocytes. Because conditioned medium from cardiac myocytes subjected to hypoxia/reoxygenation could induce extensive apoptosis of cardiac myocytes under normoxia, we thought some humoral factor released from cardiac myocytes mediated apoptosis. And, we identified the apoptosis-inducing humoral factor in the hypoxia/reoxygenation-conditioned medium by a proteomic approach. We found that eukaryotic translation initiation factor 5A (eIF5A) undergoes sulfation of 69th tyrosine residue in the *trans*-Golgi as well as more hypusination, and is rapidly secreted from cardiac myocytes in response to hypoxia/reoxygenation, then induces apoptosis of the cells as a pro-apoptotic ligand [1]. We refer to this novel post-translationally modified secreted form of eIF5A, as oxidative stress-responsive apoptosis inducing protein (ORAIP). Rat model of myocardial ischaemia/reperfusion (but not ischaemia alone) markedly increased plasma levels of ORAIP. Another oxidative stress, UV-irradiation to the heart of rats also markedly increased plasma levels of ORAIP. The apoptosis induction of cardiac myocytes by hypoxia/reoxygenation and UV-irradiation was significantly suppressed by neutralizing anti-ORAIP monoclonal antibodies (mAbs) *in vitro*. Furthermore, *in vivo* administration of anti-ORAIP mAbs significantly reduced myocardial ischaemia/reperfusion injury [1]. These data indicate that the apoptosis induction of cardiac myocytes by these stimuli is critically mediated by ORAIP. To investigate whether the role of ORAIP in apoptosis-induction is common to various types of oxidative stress, we analysed plasma levels of ORAIP in rats subjected to three different models of oxidative stress including N_2_/O_2_ inhalation, cold/warm-stress (heat shock) and blood acidification.

## MATERIALS AND METHODS

### Animal models of oxidative stress

The present study was carried out in accordance with the Guide of The Japanese Association of Laboratory Animal Facilities of National University Corporations and with the approval of Institutional Animal Care Committee. Wistar rats (male, 7 weeks old) were used in the present study. Rats were anesthetized with pentobarbital (40 mg/kg, intraperitoneally). For N_2_/O_2_ inhalation study, rats were intubated and ventilated with room air with a respirator (SN-480-7x2T; SHINANO Manufacturing). Then, they were ventilated with 100% N_2_ for 1 min immediately followed by continuous ventilation with 100% O_2_ until the end of study. For cold/warm-stress study, rats were incubated in a cold bath (4°C) for 1 min immediately followed by incubation in a warm bath (42°C) for 1 min, then transferred into a warm incubator (42°C) until the end of study. For blood acidification study, rats were injected with 1 ml of 0.1 M HCl intravenously.

### Anti-eIF5A monoclonal antibodies

A mouse anti-eIF5A mAb (clone YSP5-45-36) was generated against human eIF5A peptides (amino acid residues 44–72, which includes the hypusination site and 69th tyrosine sulfation site, coupled to KLH (keyhole limpet hemocyanin)). Another mouse anti-eIF5A mAb (clone YSPN2-74-18) was generated against human eIF5A peptides (amino acid residues 7–33, near N-terminal region, coupled to KLH) as described recently [1].

### ELISA

The sandwich ELISA was performed with YSPN2-74-18 as a capture antibody fixed on the wells of microtiter strips. Plasma samples and standards of known human recombinant-eIF5A were pipetted into the wells and incubated. After washing, horseradish peroxidase (HRP)-labelled YSP5-45-36 was added as a detection antibody and incubated. After washing, colour development was carried out by addition of a substrate solution, as described recently [1].

## RESULTS

**Figure 1:**
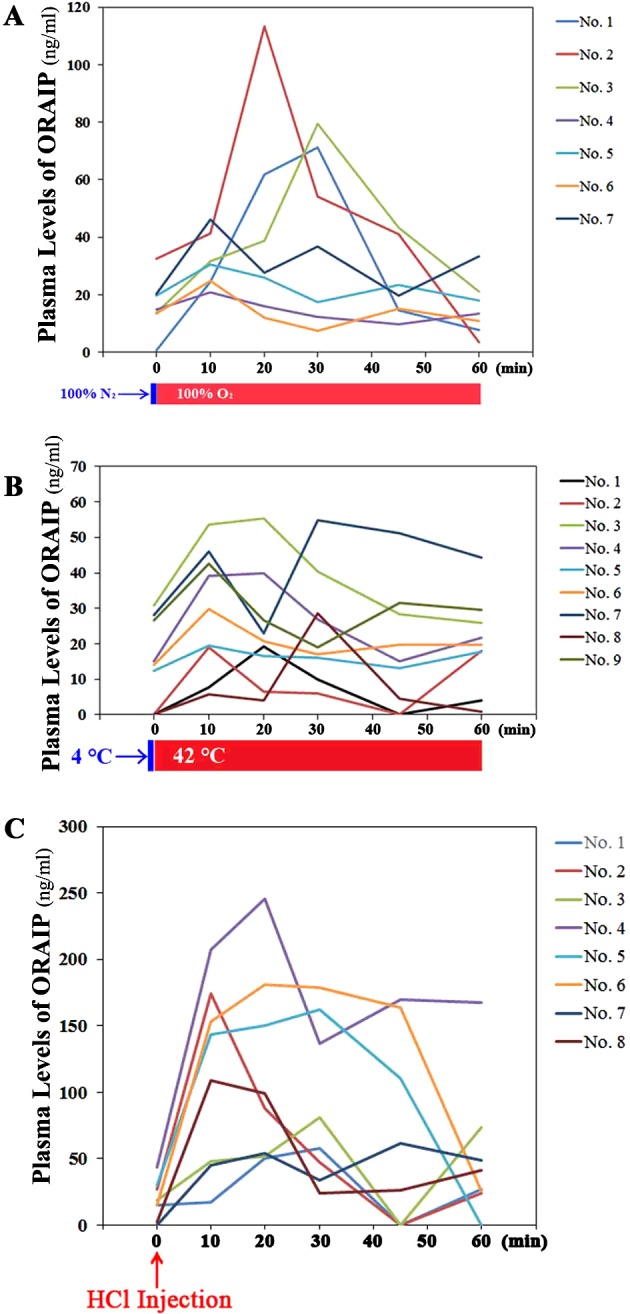
Plasma ORAIP levels in response to oxidative stresses (**A**) Plasma levels of ORAIP in rats subjected to N_2_/O_2_ inhalation (*n*=7). (**B**) Plasma levels of ORAIP in rats subjected to cold/warm-stress (*n*=9). (**C**) Plasma levels of ORAIP in rats subjected to blood acidification (*n*=8). In each experiment, plasma ORAIP concentrations significantly increased at peak levels as compared with those before stimulation [*P*=0.0014 (A), *P*=0.0001 (B), *P*=0.0013 (C); *P* values were calculated by paired *t* test].

In N_2_/O_2_ inhalation study (Figure 1A), the (mean±S.E.M.) plasma ORAIP concentrations before N_2_ inhalation were (16.4±9.6) ng/ml, they significantly increased with their peaks [55.2±34.2 ng/ml (mean±S.E.M.), *P*=0.0014, paired *t* test] at 10–30 min after the start of O_2_ inhalation. The plasma ORAIP levels clearly decreased to [15.4±9.9 ng/ml (mean±S.E.M.)] at 60 min, which were around the control levels before N_2_/O_2_ inhalation. Figure 1(B) shows the results of the second model of oxidative stress cold/warm-stress study. The (mean±S.E.M.) plasma ORAIP concentrations before cold-stress were (14.1±12.4) ng/ml, they significantly increased with their peaks [34.3±14.6 ng/ml (mean±S.E.M.), *P*=0.0001, paired *t* test] at 10–20 min after the start of warm-stress. Then, the plasma ORAIP levels decreased to [20.2±13.0 ng/ml (mean±S.E.M.)] at 60 min, but still remained higher than the control levels before cold/warm-stress. Figure 1(C) shows the results of the third model of oxidative stress blood acidification study. The (mean±S.E.M.) plasma ORAIP concentrations before intravenous HCl injection were (18.9±14.3) ng/ml, they markedly increased with their peaks [134.0±67.2 ng/ml (mean±S.E.M.), *P*=0.0013, paired *t* test] at 20–30 min after intravenous HCl injection. Then, the plasma ORAIP levels gradually decreased after 60 min, but still remained higher [51.0±51.8 ng/ml (mean±S.E.M.)] than the control levels at 60 min. To investigate how intravenous injection of 1 ml of 0.1 M HCl affects the pH of circulating blood, we analysed the pH of circulating blood of rats after HCl injection. Just before HCl injection, the (mean±S.E.M., *n*=4 at each time point) pH was (7.406±0.013). After HCl injection, the pH gradually decreased, then reached (7.192±0.145) at 60 min after HCl injection. However, there were no significant differences in the pH values among different time points (Tukey–Kramer method) up to 60 min.

## DISCUSSION

In the present study, we clearly showed for the first time that plasma levels of a novel ORAIP in rats subjected to three physicochemical models of oxidative stress were significantly increased within 30 min of stimulation. This is consistent with other models of oxidative stress such as ischaemia/reperfusion (hypoxia/reoxygenation) and UV-irradiation [1]. Because oxidative stress induced by various external stresses plays a critical role in the pathogenesis of inflammation, atherosclerosis, aging, cancer and so on [2–4], the molecular mechanism of cellular response to oxidative stress is one of the fundamental principles of biology. In a narrow sense, oxidation is defined as gain of oxygen and reduction is defined as loss of oxygen. Broadly, oxidation or reduction is defined as loss or gain of electrons (e^−^) respectively. Therefore, a substance loses electrons by oxidizing agents and various types of oxidative stress. Recently, we have identified a novel secreted form of eIF5A to be the apoptosis-inducing humoral factor in hypoxia/reoxygenation-conditioned medium of cultured cardiac myocytes [1], which we refer to as ORAIP. We confirmed that myocardial ischaemia/reperfusion (but not ischaemia alone) markedly increased the plasma levels of ORAIP *in vivo* [1], supporting that secretion of ORAIP is specific to oxidative stress. In the present study, we confirmed that ORAIP can be secreted in response to three other types of physicochemical oxidative stress *in vivo*. It is clear that ventilation with 100% N_2_ for 1 min immediately followed by continuous ventilation with 100% O_2_ induces rapid hyperoxygenation of blood, making systemic cells exposed to high concentration of O_2_. For heat shock, temperature elevation (heat shock) leads to mitochondrial membrane hyperpolarization, which in turn increases reactive oxygen species (ROS) production in wheat cells [5]. It has also been shown that heat shock can affect the redox state and induce oxidative stress in fish [6]. For mammals, hyperthermia increases glutathione peroxidase activity and ROS production, and induces apoptosis of murine spermatogenic cells [7]. Thus, oxidative stress induction by heat shock is common among different species. For blood acidification, Rustom et al. [8] reported that acidosis of cultured renal tubular epithelial cells *in vitro* leads to a reduction in glutathione and an increase in glutathione peroxidase activity and NH_3_ generation, reflecting oxidative stress. Using proximal tubular cells, Souma et al. [9] demonstrated that oleic acid-bound albumin induced ROS production, as a model for proteinuria-induced renal injury, was significantly enhanced by acidification resulting in apoptosis induction through activation of Pyk2. Pekun et al. [10] reported that acidification induces depolarization of mitochondria followed by ROS synthesis and oxidative stress in synaptosomes. Thus, acidification (lowering pH) of cellular environment causes oxidative stress to the cells.

In conclusion, these data strongly suggest that secretion of ORAIP in response to various types of oxidative stress is universal mechanism and plays an essential role in cellular response to the external stresses. ORAIP will be an important novel biomarker as well as a specific therapeutic target of these physicochemical oxidative stress-induced cell injuries as well as ischaemia/reperfusion injury.

## Abbreviations

eIF5A: eukaryotic translation initiation factor 5A
KLH: keyhole limpet hemocyanin
mAb: monoclonal antibody
ORAIP: oxidative stress-responsive apoptosis inducing protein
ROS: reactive oxygen species
